# Optimierung der Verpflegung in Kita und Grundschule: Ergebnisse eines Delphi-Verfahrens im Rahmen der Evaluation eines IN FORM-Projekts

**DOI:** 10.1007/s11553-021-00901-5

**Published:** 2021-09-15

**Authors:** Marlen Niederberger, Susanne Nowitzki-Grimm, Lisa Werner, Katja Schleicher, Petra Lührmann

**Affiliations:** Forschungsmethoden in der Gesundheitsförderung und Prävention, Pädagogische Hochschule Schwäbisch Gmünd, Oberbettringer Str. 200, 73525 Schwäbisch Gmünd, Deutschland

**Keywords:** Gesundheitsförderliche Verpflegung, Essumgebung, Informelles Lernen, Ernährungsbildung, Delphi-Verfahren, Health-promoting meals, Eating environment, Informal learning, Nutritional education, Delphi method

## Abstract

**Hintergrund:**

Im Rahmen von „IN FORM – Deutschlands Initiative für gesunde Ernährung und mehr Bewegung“ will das Projekt „Optimierung der Verpflegung in Kita und Schule“ wirksame Impulse in der Verhaltens- und Verhältnisprävention setzen und die Verknüpfung von Ernährungsbildung und Verpflegung in Kita und Grundschule voranbringen. Im Jahr 2019 gab das Landeszentrum für Ernährung Baden-Württemberg an der Landesanstalt für Landwirtschaft, Ernährung und Ländlicher Raum eine wissenschaftliche Evaluation der Umsetzung des Projekts in Baden-Württemberg in Auftrag.

**Ziel der Arbeit:**

Den Abschluss der methodenintegrativen Evaluation bildete ein Delphi-Verfahren, bei dem konsentierte Handlungsempfehlungen zur Optimierung der Verpflegung in Kita und Grundschule identifiziert wurden. Hierzu wurden Expert*innen um ihre Urteile zur Qualität des Verpflegungsangebots, zur Vernetzung relevanter Akteur*innen, zur formalen Ernährungsbildung und zur strukturellen Verankerung der Verpflegung gebeten.

**Methodik:**

Durchgeführt wurde ein dreistufiges online-basiertes Delphi-Verfahren. In zwei Befragungsrunden wurden die am Projekt beteiligten Praxisakteur*innen und externe Wissenschaftler*innen standardisiert befragt. Im Anschluss wurde ein Online-Workshop durchgeführt, bei dem dissente Urteile der vorherigen Befragungen offen mit den Expert*innen diskutiert und mögliche Hintergründe eruiert wurden.

**Ergebnisse und Diskussion:**

Die Expert*innen sind sich einig, dass eine gesundheitsförderliche Kita- und Schulverpflegung für das informelle Lernen wichtig ist und mit der formalen Ernährungsbildung verknüpft werden sollte. Für die Umsetzung sehen sie eine zentrale Rolle bei Kita- bzw. Schulleitungen und festen Ansprechpersonen in den jeweiligen Einrichtungen. Einen Handlungsbedarf sehen die Expert*innen in der Sensibilisierung, Beteiligung und Schulung verschiedener Akteur*innen sowie im Ausbau der Unterstützungsangebote für Kita und Grundschule.

## Abstract (en)

**Background:**

As part of “IN FORM—Germany’s initiative for healthy nutrition and more exercise”, the project “Optimizing catering offer in daycare centers and schools” aims to provide effective impulses in behavioral and environmental prevention and to advance the linking of nutritional education with catering offer (meals) in daycare centers and elementary schools. A scientific evaluation of the project in Baden-Württemberg, Germany was commissioned in 2019 by the Regional Center of Nutrition Baden-Württemberg at the State Authority for Agriculture, Nutrition and Rural Affairs Baden-Württemberg, Germany.

**Objectives:**

The method-integrative evaluation was concluded with a Delphi process in which consensual recommendations for action to optimize the catering offer in daycare centers and elementary schools were identified. For this purpose, experts were asked for their opinions on the quality of the catering offer, the networking of relevant actors, formal nutritional education and the structural integration of the catering offer (informal learning).

**Materials and methods:**

A three-stage online Delphi method was carried out. In two survey rounds, the experts (practitioners involved in the project and external scientists) were interviewed in a standardized way. This was followed by an online workshop in which dissenting opinions from the previous surveys were openly discussed with the experts and possible backgrounds were investigated.

**Results and conclusion:**

The experts agree that a health-promoting catering offer in daycare centers and schools is essential for informal learning in the field of nutrition and should be linked to formal nutritional education. For the implementation, they see a central role in the daycare and school management and fixed contact persons in the respective facilities. The experts see a need for action in sensitization, participation, and training of various actors as well as in the expansion of support services for daycare centers and elementary schools.

Kinder und Jugendliche in Deutschland weisen häufig ein ungünstiges Ernährungsverhalten auf, wobei Heranwachsende aus Familien mit einem niedrigen soziökonomischen Status besonders betroffen sind [20]. Kitas und Grundschulen bilden Settings, in denen eine gesundheitsförderliche Ernährung aller Kinder unabhängig vom soziokulturellen Hintergrund unterstützt werden kann. Im Rahmen einer Evaluation eines IN FORM-Projekts haben Expert*innen aus Praxis und Wissenschaft Handlungsempfehlungen zur Optimierung der Verpflegung in Kita und Grundschule formuliert.

Im Kindes- und Jugendalter sind eine gesundheitsförderliche Verpflegung sowie eine fundierte Ernährungsbildung von großer Bedeutung. Dahinter steckt die Einsicht, dass Ursachen von Morbidität, Mortalität und gesundheitlicher Beeinträchtigung reduziert werden können, wenn ernährungsbezogene Verhaltensweisen, die sich in Kindheit und Jugend etablieren und durch soziale, kulturelle sowie politische Rahmenbedingungen geprägt werden, günstig beeinflusst werden [13]. Im Zuge des Ausbaus und der vermehrten Inanspruchnahme der Ganztagsangebote sind im Kontext von Verpflegung und Ernährungsbildung Kitas und Grundschulen vermehrt in den Fokus dieser Diskussion gerückt [2, 15]. So lag der Anteil an Grundschulen mit Ganztagsbetrieb im Schuljahr 2018/2019 bei 68 %, d. h. 42 % der Kinder besuchen eine entsprechende Grundschule und die Tendenz ist steigend [21].

Studien belegen, dass die Settings Kita und Schule durch ein Zusammenspiel verhaltens- und verhältnisbezogener Maßnahmen große Chancen für die ernährungsbezogene Gesundheitsförderung bieten [10, 13]. Voraussetzungen sind u. a., dass sich Aktivitäten an die Kinder richten und weniger an die Eltern, alle relevanten Akteursgruppen (u. a. pädagogische Fachkräfte bzw. Lehrkräfte, Leitungen) einbezogen und sozialräumliche Besonderheiten beachtet werden. Aus Sicht der Ernährungsbildung spielen hier die formale Ernährungsbildung, das informelle Lernen in alltäglichen Lebenszusammenhängen sowie die Verzahnung beider zu einem gemeinsamen pädagogischen Konzept eine besondere Rolle (Tab. 1, [15]). Doch die Umsetzung ist noch nicht flächendeckend gelungen, wie folgende Entwicklungen verdeutlichen:

- Die Themen Essen und Ernährung sind in den Orientierungs- und Bildungsplänen der Bundesländer für Kita und Schule verankert, jedoch sehr unterschiedlich realisiert. Bedeutsame Inhalte, wie Essen oder der Umgang mit Lebensmitteln, werden selten bis gar nicht bzw. nur in Form von sporadisch stattfindenden Projekten thematisiert [2, 12].
- Pädagogische Fachkräfte in Kitas und Schulen sind durch ihre Ausbildung häufig unzureichend für den Bildungsbereich Essen und Ernährung qualifiziert [12].
- Das Verständnis von Ernährungsbildung von Kitaleitungen ist heterogen und nicht immer differenziert. Gerade einmal 12 % der Kitaleitungen einer bundesweiten Befragung geben an, über ein umfassendes Verständnis von Ernährungsbildung zu verfügen [12].
- Aktuelle Untersuchungen weisen teilweise erhebliche Diskrepanzen zwischen dem Lebensmittelangebot in der Kita- und Schulverpflegung und den Empfehlungen der Deutschen Gesellschaft für Ernährung e. V. (DGE) auf [1, 2].
- Bisher sind nur wenige Schulen nach dem DGE-Qualitätsstandard für die Schulverpflegung zertifiziert, obwohl die Zertifizierung ein erklärtes politisches Ziel aller Bundesländer ist [2, 7].
- In Kitas und Schulen fehlen oftmals geeignete separate Speiseräume [2, 3]. So zeigt eine bundesweite Studie mit 1408 Kitas, dass die Mittagsmahlzeit in knapp 80 % der Einrichtungen in einem Gruppenraum eingenommen wird. In einer bundesweiten Erhebung zur Qualität der Schulverpflegung bewertete ein Großteil der Schüler*innen die Geräuschkulisse und die Gemütlichkeit des Speiseraums eher schlecht [2].
- Kinder und Jugendliche werden bei der Planung und Umsetzung des Verpflegungsangebots und der Rahmbedingungen nicht ausreichend beteiligt. So geben nur 60 % der Kitas in Deutschland an, dass die Geschmacksvorlieben der Kinder bei der Planung der Speisen berücksichtigt werden [3]. In Grundschulen in Deutschland werden nur 21 % der Schüler*innen an der Gestaltung des Mittagsessens beteiligt [2].

**Tab. 1:** Zugrunde liegende Definitionen des Delphi-Prozesses

Ernährungsbildung	Formale Ernährungsbildung	Informelles Lernen
Zielt darauf ab, Menschen zu befähigen, die eigene Ernährung politisch mündig, sozial verantwortlich und demokratisch teilhabend unter den komplexen gesellschaftlichen Bedingungen zu gestalten. Die moderne kompetenzorientierte Ernährungsbildung stellt dabei den essenden Menschen – und nicht Energie und Nährstoffe – in den Mittelpunkt. In der Kita und Schule ist der Erwerb von Kompetenzen in der Auseinandersetzung und Reflexion der eigenen Ernährungsweise zentral [4, 15]. Dabei wird unterschieden zwischen formaler Ernährungsbildung und informellem Lernen.	Ist in Bildungs- und Ausbildungseinrichtungen wie Kita und Schule institutionell verankert und findet planvoll statt. In den Orientierungs- und Bildungsplänen der Bildungsinstitutionen werden Lerninhalte, Lernziele, Lernprozesse und die Lernorganisation verbindlich festgelegt. Formale Bildungsprozesse führen zu anerkannten Abschlüssen.	Im Bereich von Essen und Trinken wird als nicht didaktisch organisiertes Lernen in alltäglichen Lebenszusammenhängen begriffen, das von den Lernenden nicht immer als Erweiterung ihres Wissens und ihrer Kompetenzen wahrgenommen wird. Es findet eher zufällig statt und kann sowohl innerhalb als auch außerhalb von formalen Bildungsinstitutionen zustande kommen. In Kita und Schule finden sich zahlreiche Anknüpfungspunkte für das informelle Lernen, z. B. bei der Verpflegung [4, 15].

## Evaluation eines IN FORM-Projekts

Im Rahmen des Nationalen Aktionsplans „IN FORM – Deutschlands Initiative für gesunde Ernährung und mehr Bewegung“ wurde das Projekt „Optimierung der Verpflegung in Kita und Schule durch Intervention bei Anbietern und Einrichtungen“ vom Landeszentrum für Ernährung Baden-Württemberg von Mai 2019 bis Dezember 2020 durchgeführt. Ziel ist es, im Bereich der Gemeinschaftsverpflegung und Ernährungsbildung wirksame Impulse für die ernährungsbezogene Verhaltens- und Verhältnisprävention zu setzen [14]. Hierzu bietet das Landeszentrum für Ernährung Baden-Württemberg Coachings von Caterern, Kitas und Grundschulen sowie darin integriert verschiedene Vernetzungsformate an.

Mit Unterstützung einer externen Evaluation [16] wurden das Konzept, die Durchführung und die Wirkung analysiert. Auf der Grundlage des aktuellen Forschungsstandes und der Ergebnisse der methodenintegrativen Evaluation (Dokumentenanalyse der Kommunikationsformate, Befragungen der Zielgruppen zum Coaching, Auswertung von Speiseplänen) wurden mittels eines Delphi-Verfahrens auf einer übergeordneten Ebene Handlungsempfehlungen zu folgenden Themenblöcken in Kita und Grundschule fokussiert:

1. Qualität des Verpflegungsangebots,
2. Vernetzung der Akteur*innen,
3. formale Ernährungsbildung,
4. strukturelle Verankerung des Themas Verpflegung in Kita- und Schulalltag.

## Methodik

Die Identifikation der Handlungsempfehlungen erfolgte durch ein Delphi-Verfahren. Dies ist ein systematisches, mehrstufiges Befragungsverfahren, bei dem Expert*innen (die am Projekt beteiligten Praxisakteur*innen und externe Wissenschaftler*innen) anhand eines standardisierten Fragebogens (basierend auf den ersten Ergebnissen der Projektevaluation und den aktuellen Befunden aus der Literatur) Einschätzungen und Bewertungen zu einem bestimmten Thema abgeben.

Das Besondere des Delphi-Verfahrens ist, dass bei jeder erneuten Befragung die statistischen Befunde der vorherigen Befragung mit angegeben werden und so die Befragten ihre Urteile überdenken und ggf. revidieren können. Dieses Verfahren hat sich bewährt, wenn es um die Formulierung von konsentierten Handlungsempfehlungen von Expert*innen zu einem klar abgrenzbaren Thema geht [18].

In der vorliegenden Projektevaluation wurde das Delphi-Verfahren über drei Runden als Online-Format durchgeführt. Ziel war die Ermittlung von Konsens zu den Handlungsempfehlungen für die Entwicklung eines ganzheitlichen Ernährungskonzepts für Kitas und Grundschulen in Baden-Württemberg. Deshalb wurde das Delphi-Verfahren am Ende zur Zusammenfassung und Synthese der vorhergehenden Analysen eingesetzt. Verwendet wurden v. a. fünf- und sechsstufige Ratingskalen. Zudem wurde bei jedem Item auf einer vierstufigen Ratingskala erfragt, wie sicher sich die Expert*innen bei der Antwort sind. Konsens wurde statistisch definiert:

- sechsstufige Ratingskala: Standardabweichung < 1,0 und Modus bei der Urteilssicherheit „sicher“ bzw. „sehr sicher“,
- fünfstufige Ratingskala: Standardabweichung < 0,9 und Modus bei der Urteilssicherheit „sicher“ bzw. „sehr sicher“,
- Auswahl der wichtigsten Aspekte: Stabilität der Urteile von der ersten zur zweiten Runde.

Konsens heißt also nicht, dass alle Expert*innen aus Praxis und Wissenschaft die gleiche Meinung bzw. Beurteilung teilen müssen. Vielmehr wurde eine Annäherung in den Urteilen angestrebt. Konkret wurden drei aufeinanderfolgende Arbeitsschritte durchgeführt:

1. Erste Online-Befragung der Expert*innen (erste Runde): Formuliert wurden standardisierte und offene Fragen. Die standardisierten Items basieren auf den Ergebnissen der vorherigen Analysen im Rahmen der Projektevaluation und spiegeln den aktuellen Wissensstand zum Thema Ernährung in Kita und Grundschule wider. Der Fragebogen wurde über *unipark *programmiert. Die Auswertung erfolgte mit dem Statistikprogramm SPSS (IBM SPSS Statistics 27). Die standardisierten Daten wurden deskriptiv (Mittelwerte und Standardabweichungen) und die offenen Antworten qualitativ-inhaltsanalytisch ausgewertet [17].
2. Zweite Online-Befragung der Expert*innen (zweite Runde): Die Befragten erhielten erneut einen Online-Fragebogen inklusive der aggregierten Gruppenantworten und ihrer individuellen Antworten der ersten Runde. Die Expert*innen hatten so die Möglichkeit, ihre Urteile zu überdenken und ggf. zu revidieren. Aus den offenen Fragen der ersten Runde wurden standardisierte Items generiert. Urteile, die bereits nach der ersten Runde im Konsens waren, wurden nicht wieder aufgenommen.
3. Online-Workshop unklarer bzw. dissenter Befunde mit Expert*innen (dritte Runde): Der Workshop wurde aufgrund der COVID-19-Pandemie („coronavirus disease 2019“) als Online-Format realisiert. Zentrales Anliegen war es, inhaltliche Begründungen für dissente Urteile zu erfassen.

## Stichprobe

**Abb. 1:**
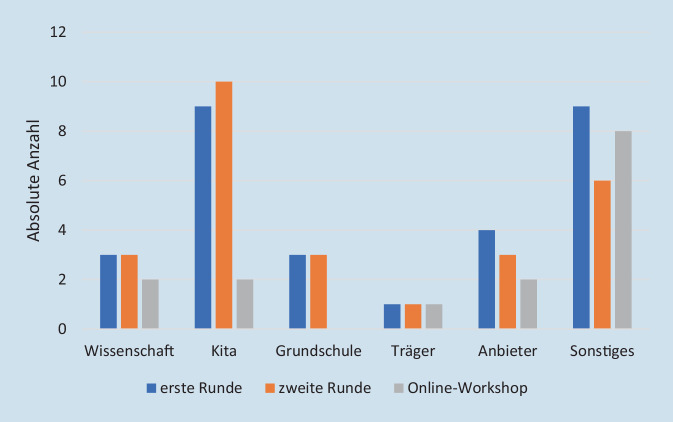
Delphi-Verfahren: teilnehmende Expert*innen. (Anmerkung: Unter *Sonstiges* wurde angegeben: Beratung, Coach, Coachin, Erwachsenenbildung, öffentlicher Gesundheitsdienst)

Zur Teilnahme am Delphi-Prozess wurden alle am Projekt beteiligten Praxisakteur*innen, d. h. Caterer, Coach*innen, Beteiligte aus Kitas, Schulen, Kommunen und Träger (*n* = 30) sowie externe Wissenschaftler*innen aus den Bereichen Verpflegung und Ernährungsbildung (*n* = 6) eingeladen. Insgesamt haben 29 Personen bei der ersten Befragungsrunde, 26 Personen bei der zweiten Befragungsrunde und 14 Personen beim Online-Workshop mitgemacht. Es ist gelungen, in allen drei Runden des Delphi-Prozesses die Bandbreite an Expertise zu integrieren (Abb. 1). Dabei ist zu beachten, dass einige Teilnehmende Expertisen in verschiedenen Bereichen hatten. So begleiten beispielsweise einige der Coach*innen Kitas und Schulen. Die Integration von Kindern und Eltern war aufgrund des kognitiven und zeitlichen Anspruchs an die Befragten sowie möglicher verzerrender Gruppeneffekte beim Online-Workshop in diesem Delphi-Verfahren nicht vorgesehen.

## Ergebnisse

Im Folgenden werden die konsentierten Urteile der Expert*innen beschrieben (Tab. 2). Dabei werden die inhaltlichen Argumente des Online-Workshops integriert.

**Tab. 2:** Überblick konsentierte Handlungsempfehlungen und Unterstützungsbereiche für Kitas und Grundschulen^a^

	Konsentierte Handlungsempfehlungen	Wichtigste Unterstützungsbereiche
Qualität des Verpflegungsangebots	Notwendigkeit einer regelmäßigen IST-Analyse	IST-Analyse
	Umsetzung der DGE-Qualitätsstandards	Reduzierung von vermeidbaren Lebensmittelverlusten
	Beteiligung von Eltern und Essensanbietern	Schnittstellenmanagement und Kommunikation
	Beachtung einer angemessenen Essatmosphäre	Anpassung der Angebote an die Bedürfnisse von Kindern
	Schaffung eines kostenneutralen gesundheitsförderlichen Verpflegungsangebots	
Vernetzung der Akteur*innen	Beteiligung aller relevanten Akteur*innen	Durchführung von Fachtagungen, Fortbildungen und Workshops
	Zentrale Gelingensfaktoren sind feste Ansprechpersonen in den Einrichtungen, Unterstützung durch die Leitung, Verankerung des Themas Verpflegung in Konzeption, Transparenz und Bereitstellung von Informationen für Eltern	Prozessbegleitung, -moderation und -dokumentation
	Etablierung eines kommunalen, institutionsübergreifenden Verpflegungsausschusses	
Formale Ernährungsbildung	Wichtigste Themen sind: Wertschätzung von Lebensmitteln, Nahrungszubereitung, Kompetenzen der Kinder sowie außerschulische Lerngänge	Kooperation mit außerschulischen Akteur*innen
		Ideen für praktische Aktivitäten
	Notwendigkeit der Sensibilisierung und Schulung pädagogischer Fachkräfte	Weiterbildungsangebote für Fachkräfte
	Ernährungsbildung als eine Gemeinschaftsaufgabe etablieren	Ideen für die Nahrungszubereitung mit Kindern
Strukturelle Verankerung des Themas Verpflegung in Kita- und Schulalltag	Verknüpfung von formaler Ernährungsbildung und Verpflegung (informales Lernen)	Kooperation mit außerschulischen Akteur*innen
	Verankerung der gesundheitsfördernden Schule als Grundsatz der Schulpolitik	Weiterbildungsangebote für Fachkräfte
		Anknüpfung an die Orientierungs- und Bildungspläne
		Personelle Unterstützung

*DGE* Deutsche Gesellschaft für Ernährung e. V.

^a^Die Rangfolge der Unterstützungsbereiche entspricht der Rangfolge der Expert*inneneinschätzung, angegeben sind jeweils die am häufigsten genannten Bereiche

## Qualität des Verpflegungsangebots

Um dauerhaft hochwertige Verpflegungsangebote in Kita und Grundschule zu gewährleisten, sind nach Ansicht der Expert*innen regelmäßige IST-Analysen notwendig. Sie sind überzeugt, dass eine gesundheitsförderliche Verpflegung für Eltern ein wichtiges Thema ist und diese auch bereit wären, entsprechende Maßnahmen zu unterstützen. Sie zweifeln, ob die Eltern eine Preiserhöhung akzeptieren würden. Die Expert*innen sehen allerdings die Möglichkeit eines kostenneutralen Angebots und zwar durch Umstrukturierungsmaßnahmen bei Caterern sowie die Reduzierung von Lebensmittelabfällen. Die Expert*innen plädieren zudem für soziale Ausgleichsmechanismen, um ein gesundheitsförderliches Verpflegungsangebot für alle Kinder bereitstellen zu können. Eine faire Lastenverteilung, Chancengleichheit und die Berücksichtigung von Kinderrechten sei auch beim Thema Verpflegung unabdingbar. Hier sei v. a. die Politik gefragt, eine gerechte Lösung zu finden.

Über eine verpflichtende Umsetzung der DGE-Qualitätsstandards [5, 6] in Kita und Grundschule konnte in den standardisierten Befragungen kein Konsens erreicht werden. Im Workshop wurde deutlich, dass hier eine Differenzierung zwischen Umsetzung und Zertifizierung notwendig ist. Die Umsetzung der DGE-Qualitätsstandards wird für wichtig erachtet und solle auch in den Leitlinien bzw. Konzeptionen der Einrichtungen verankert werden. Die Zertifizierung hingegen stellt einige Einrichtungen vor große Herausforderungen und werde mitunter aufgrund der strikten Vorgaben als „Gesundheitsdiktatur“ empfunden. Regionale und strukturelle Spezifika können eine Zertifizierung erschweren oder erleichtern und müssen daher berücksichtigt werden. Den Expert*innen zufolge hat eine angenehme Essatmosphäre eine große Bedeutung für die ernährungsbezogene Gesundheitsförderung.

Zur Gewährleistung der Qualität des Verpflegungsangebots benötigen Kitas und Grundschulen v. a. bei der IST-Analyse, der Reduzierung von Lebensmittabfällen, im Bereich Schnittstellenmanagement und Kommunikation sowie bei der Planung und der Anpassung der Angebote an die Bedürfnisse von Kindern externe Unterstützung.

## Vernetzung der Akteur*innen

Die Expert*innen sind sich einig, dass die Träger, die Kita- bzw. Schulleitung, die pädagogischen Fachkräfte, das Verpflegungsteam, die Verpflegungsanbieter, die Essensgäste und die Eltern bei der Optimierung des Verpflegungsangebots einzubeziehen und zu vernetzen sind. Um dies zu gewährleisten, müssen relevante Informationen transparent gemacht, feste Ansprechpersonen in den Einrichtungen benannt, die Unterstützung durch die Kita- bzw. Schulleitung sichergestellt und das Thema Verpflegung im Leitbild bzw. der Konzeption verankert werden.

Als erfolgsversprechendes Format der Vernetzung schlagen die Expert*innen einen institutionsübergreifenden Verpflegungsausschuss auf kommunaler Ebene vor. Zu den zentralen Aufgaben gehöre es, alle auf denselben Wissenstand zu bringen und sich gegenseitig zu informieren. Auch ein Beschwerdemanagement, die Initiation von Optimierungsprozessen, die Mitwirkung bei Aktionen, die Unterstützung bei IST-Analysen bzw. Evaluationen, die Öffentlichkeitsarbeit, die Mitwirkung bei Ausschreibungen (Anbieterauswahl) und die Festlegung von Standards seien wichtige Aufgaben. Neben den genannten Akteur*innen könnten bei Bedarf weitere Akteur*innen wie Facility-Manager*innen oder Vertreter*innen des Gesundheitsamts eingebunden werden.

Für eine gelingende Vernetzung benötigen Kitas und Grundschulen Unterstützung in Form von Fortbildungen, Fachtagungen sowie eine externe Prozessbegleitung bzw. -moderation.

## Formale Ernährungsbildung

Ernährungsbildung gehört für die Expert*innen zur Grundbildung und ist unverzichtbar. Sie verstehen sie als eine Gemeinschaftsaufgabe, bei der Eltern und pädagogische Fachkräfte gemeinsam agieren. Die momentane Ausgestaltung der Ernährungsbildung in Kita und Grundschule hängt aber mitunter von dem Engagement einzelner Personen ab. Deshalb sei es notwendig, pädagogische Fachkräfte für diese Thematik zu sensibilisieren und zu schulen. Zu den zentralen Themen der formalen Ernährungsbildung in Kita und Grundschule gehören Wertschätzung von Lebensmitteln, Nahrungszubereitung sowie Kompetenzen der Kinder zur Reflexion der eigenen Ernährungsweise. Auch außerschulische Lerngänge halten die Expert*innen zentral für eine gelingende formale Ernährungsbildung.

Kitas und Grundschulen brauchen in diesem Bereich v. a. Ideen für praktische Aktivitäten und Anregungen für die Nahrungszubereitung mit den Kindern. Sie benötigen Weiterbildungsangebote und die Kooperationen mit außerschulischen Akteur*innen müssen ausgebaut werden.

## Strukturelle Verankerung des Themas Verpflegung in Kita- und Schulalltag

Die Expert*innen sind sich einig über die Notwendigkeit der Verknüpfung von formaler Ernährungsbildung und Verpflegung (informelles Lernen) zu einem Gesamtkonzept. Dies kann durch die Sensibilisierung und Schulung der pädagogischen Fachkräfte, den gemeinsamen Mahlzeiten von pädagogischen Fachkräften und Kindern, einer gesundheitsförderlichen Gestaltung des Essensumfelds sowie im Kontext der Grundschule der Verankerung der gesundheitsfördernden Schule als Grundsatz in der Schulpolitik erfolgen.

Unterstützung brauchen Kitas und Grundschulen insbesondere beim Aufbau von Kooperationen mit außerschulischen Akteur*innen. Zudem sind ernährungsbezogene Weiterbildungsangebote und konkrete Ideen für die Umsetzung, insbesondere die Anknüpfung an die Orientierungs- und Bildungspläne und eine personelle Unterstützung notwendig.

## Diskussion

Als Barrieren in Kitas und Grundschulen für ein optimiertes Verpflegungsangebot werden in verschiedenen Studien u. a. fehlende Ressourcen, die mangelnde Vernetzung und Kommunikation der relevanten Akteur*innen sowie das Wissen und das Bewusstsein für die Chancen der Verpflegung und Ernährungsbildung, das Engagement der beteiligten Akteur*innen sowie das Aufbrechen von Ernährungsgewohnheiten bei den Kindern identifiziert [2, 3, 12]. Diese Erkenntnisse bestätigt auch die vorliegende Evaluationsstudie. Die Handlungsempfehlungen der befragten Expert*innen des Delphi-Prozesses setzen daher genau an diesen Punkten an. Sie verweisen insbesondere auf die Relevanz von Weiterbildungen, die Unterstützung bei der praktischen Umsetzung und dem Aufbau von Kooperationen. Zudem fordern sie, ähnlich wie in anderen Studien [1–3] eine verbindliche Umsetzung der DGE-Qualitätsstandards für die Verpflegung in Kita und Schule. Jedoch bleibt die Art der Umsetzung offen. Die Zertifizierung kann allerdings nach Ansicht der Expert*innen nicht verpflichtend eingefordert werden, weil die Bedingungen und Kontexte in den jeweiligen Einrichtungen zu verschieden sind.

Themenübergreifend gilt es, die strukturellen und formalen *Besonderheiten von Kitas und Grundschulen* zu beachten. Die Themenfelder und Erwartungen in der Grundschule sind deutlich umfassender und in der Bewertung der befragten Expert*innen heterogener als in der Kita. Zudem werde das Thema Verpflegung in der Grundschule oftmals noch nicht als pädagogische Aufgabe wahrgenommen bzw. die Bildungspotentiale des Verpflegungsangebots nicht erkannt.

Die Diskussion während des Online-Workshops zeigt deutlich, dass Kitas und Grundschulen bedarfsgerechte und vielfältige Unterstützungsangebote brauchen. Landesinstitutionen, wie in dem vorliegenden Projekt das durchführende Landeszentrum für Ernährung Baden-Württemberg, können hier nach Ansicht der Expert*innen auf vielfältige Weise wirken. Dazu gehören insbesondere die Schaffung von Möglichkeiten zur Vernetzung der Akteur*innen und eine Rückkopplung an das zuständige Ministerium, z.B. wie elementar wichtig die Bereitstellung von Ressourcen zur Etablierung günstiger struktureller Rahmenbedingungen in den Einrichtungen ist. Nur so kann die nach Ansicht der Expert*innen aktuell größte Herausforderung, nämlich die verbindliche und nachhaltige Verankerung des Themas Verpflegung im Kita- und Grundschulalltag, angegangen werden. Bei der Sensibilisierung von politischen Entscheidungsträger*innen spielten derartige Institutionen daher eine zentrale Rolle. Diese verschiedenen Angebote seien nach Ansicht der befragten Expert*innen notwendig, um letztendlich allen Kindern – unabhängig von ihrer sozialen Lage – Zugang zu einer gesundheitsförderlichen Verpflegung und Ernährungsbildung zu ermöglichen.

Schließlich gibt es *sozialräumliche Disparitäten* zwischen den Einrichtungen, die es zu beachten gilt und die spezifisch zugeschnittene Unterstützungsstrukturen notwendig machen, wie in dem Projekt beispielsweise in Form von Coachings umgesetzt. Etwa ein Viertel bis ein Drittel der Grundschulen in Deutschland ist in Städten bzw. Landkreisen mit einer überdurchschnittlich hohen Armutsbelastung und/oder einem hohen Anteil an Migrant*innen lokalisiert [22]. Diese „Verräumlichung sozialer Ungleichheit“ [9] hat für Kitas und Schulen in benachteiligter Lage insofern eine zentrale Bedeutung, als dass sie mit einer Kumulation ungünstiger Kompositionsmerkmale der Heranwachsenden einhergeht [8, 19] und entsprechend der Etablierung gesundheitsfördernder Kitas bzw. Grundschulen eine besonders hohe Relevanz zukommt. Möglicherweise brauchen gerade diese Einrichtungen spezifische Unterstützungsangebote, insbesondere beim Thema Vernetzung und Kooperation. Dies ist auch wichtig, weil pädagogische Fachkräfte im Bereich der Ernährungsbildung oftmals nicht ausreichend ausgebildet sind [12].

Zu vermuten ist, dass die Relevanz dieser beiden Settings für die Verpflegung und Ernährungsbildung nach der *COVID-19-Pandemie* noch wichtiger wird, weil viele Studien die zunehmende soziale Spaltung bestätigen [23] und auf kritische Entwicklungen im Bereich Bildung und Gesundheit verweisen [11]. So haben insbesondere sozial benachteiligte und leistungsschwache Schüler*innen in der Pandemiezeit zugenommen, bewegen sich weniger und konsumieren mehr digitale Medien [11, 23].

## Limitationen

Es ist zu beachten, dass sich die Studie auf Kitas und Grundschulen in Baden-Württemberg bezieht. In diesem Bundesland ist die Mittagsverpflegung eher teuer und die Inanspruchnahme im Vergleich zu anderen Bundesländern eher gering [1, 3]. Zudem fand in dem Delphi-Prozess keine Beteiligung der zentralen Zielgruppe der Kinder und ihrer Eltern statt. Die Differenzierung nach den Settings Kita und Grundschule wäre interessant gewesen, ließ sich aber aufgrund der zeitlichen Limitierung im Online-Workshop nicht realisieren.

## Fazit für die Praxis

- Zur Optimierung des Verpflegungsangebots und der Ernährungsbildung gilt es die strukturellen Unterschiede von Kitas und Grundschulen zu beachten.
- Für eine gesundheitsförderliche Verpflegung und Ernährungsbildung ist es notwendig, multiple Akteur*innen zu beteiligen, zu sensibilisieren und zu schulen sowie konkrete Ansprechpersonen zu benennen.
- Notwendig ist eine Berücksichtigung der jeweiligen sozialräumlichen und innerbetrieblichen Strukturen sowie der pädagogischen Ziele der Einrichtungen, um passgenaue Unterstützungsangebote anbieten zu können und die Chance zur dauerhaften Etablierung zu erhöhen.
- Verpflegungsangebote und formale Ernährungsbildung sollten Teil eines Gesamtkonzepts sein, mit entsprechender Verankerung im Leitbild der Einrichtung. Damit wird die gesundheitsförderliche Verpflegung zur Chefsache erklärt.
